# Adherence of SARS-CoV-2 Delta variant to a surgical mask and N95 respirators

**DOI:** 10.2144/fsoa-2022-0025

**Published:** 2022-08-08

**Authors:** Ana C Lorenzo-Leal, Selvarani Vimalanathan, Horacio Bach

**Affiliations:** 1 Faculty of Medicine, Division of Infectious Diseases, University of British Columbia, Vancouver, BC, V6H 3Z6, Canada; 2 Pathology & Laboratory Medicine, University of British Columbia, Vancouver, BC, V6H 3Z6, Canada

**Keywords:** coronavirus, COVID-19, facial protection, mask, respirator, SARS-CoV-2

## Abstract

The use of facial protection has been adopted globally due to the COVID-19 pandemic. We dissected four respirators and one surgical mask into layers to determine whether or not the virus adheres to them. These individual layers were contaminated with the SARS-CoV-2 Delta variant, and its release by vortexing was performed. Samples were used to infect Vero cells, and a plaque assay was used to evaluate the adherence of the virus to the layers. Results showed that a cumulative log reduction of the layers reduced the load of the virus by at least sixfold. Our study confirms the effectiveness of facial protection in reducing the transmission and/or infection of SARS-CoV-2.

## Plain language summary

The use of facial protection, including masks and respirators, has been adopted globally due to the COVID-19 pandemic. These products have been demonstrated to be effective in reducing the transmission of the virus. This study tested whether the virus can adhere to the different layers of respirators and a surgical mask. These individual layers were contaminated with the COVID-19 Delta variant, and we determined the degree of release after applying a mechanical force. Samples were used to infect cells, and the adherence of the virus to the layers was assessed. Results showed that a significant reduction in the number of viruses was observed. Our study confirms the effectiveness of facial protection in reducing the transmission and or infection of SARS-CoV-2.

The pandemic of the novel coronavirus SARS-CoV-2, the causative agent of COVID-19, was initially identified in Wuhan, China, and has spread worldwide. According to the WHO, as of 15 January 2022, there have been 5,518,343 deaths and 318,648,834 confirmed cases of COVID-19 [1].

COVID-19 is spreading through airborne transmission via aerosol. This occurs mainly when breathing, speaking, sneezing, coughing or talking [2]. These transmissions are facilitated by droplets (>5 to 10 μm) and/or aerosols (≤5 μm) containing the virus SARS-CoV-2 [3]. Furthermore, aerosol accumulation, also known as the ‘gas cloud’, could remain infectious for hours in indoor spaces [3]. Thus, implementing masks or respirators significantly reduces aerosol transmission by the physical protection [4–7].

The terms respirators and medical masks are used as synonyms, but there are differences according to their use [8]. In the case of medical masks, the transmission of microorganisms from the user to the environment (inside out) is prevented. In contrast, the use of respirators protects the respiratory tract of the user against not only microorganisms but also dust, droplets, etc.

According to the US National Institute for Occupational Health and Safety (NIOSH), there are nine categories of air respirators. These categories include N (not resistant to oil, 95, 99 and 100), P (strongly resistant to oil, 95, 99 and 100) and R (somewhat resistant to oil, 95, 99 and 100). The values 95, 99 and 100 indicate the minimum filtering efficiency (in percentages) [9].

There are different types of masks or respirators with various levels of protection, which can be reusable or disposable. Generally, masks do not fit as tightly as respirators. For example, N95 respirators are classified by NIOSH as air filtration, filtering at least 95% of airborne particles. N95 respirators and surgical masks are the most common disposable masks, while reusable masks are mainly used in industrial setups with half or full facepiece respirators [7].

According to the CDC and the NIOSH, surgical masks are approved by the US FDA and intended to use as protection for the wearer against multiple hazardous environments, such as those created by sprays, dangerous fluids, splashes and droplets. However, masks would not always confer respiratory protection because of their lower reliable level of filtration against smaller particles that could be inhaled, such as airborne particles.

Surgical masks contain mainly three-layer structures with different functions. For example, the outer layer repels water, the middle layer functions as a trap of particles and prevents the penetration of microorganisms, and the inner layer absorbs the moisture [4,10].

On the other hand, N95 respirators are defined by NIOSH as a filtration system that retains at least 95% of different airborne sizes particles (from aerosols to large droplets), reducing the exposure to the user [11]. Respirators may contain extra layers compared with surgical masks. For example, N95 respirators have between three and five layers, comprising an inner and outer layer, a filter layer and in some cases, a support layer [10].

The penetration or filtration mechanism of aerosols containing SARS-CoV-2 in masks has been studied and reported [4,5,10,12,13]. Moreover, contamination with SARS-CoV-2 on the masks’ outer surface has also been reported [14]. Still, a detailed investigation has not been reported on whether the virus remains adherent to the layers. Then, this study aimed to evaluate the adherence of the SARS-CoV-2 Delta variant on different layers of N95 respirators. In addition, for comparison purposes, we assessed the adherence of the virus on a surgical mask.

## Materials & methods

### Virus & cell lines

SARS-CoV-2 Delta variant (B.1.617.2) was provided by the British Columbian Centre for Disease Control Public Health Laboratory, BC, Canada. Vero E6 cells (ATCC CCL-81) were used to replicate the virus and perform the infections. Cells were grown in minimum essential media (MEM) (Invitrogen, MA, USA) supplemented with 5% fetal calf serum (Invitrogen), pyruvate and nonessential amino acids. All the incubations were performed in an incubator at 37°C supplemented with 5% CO_2_. All the work performed in this study was conducted in level 3 at the Facility for Infectious Disease and Epidemic Research (FINDER, University of British Columbia, BC, Canada).

Viral stocks were prepared by infecting 80% confluent Vero E6 cells with 1 ml of the virus at a 5x10^5^ PFU/ml concentration. After 72 h, the supernatant was collected and cleared by centrifugation at 3200 × g for 10 min. The supernatant was aliquoted and kept at -80°C until further use. The titer of the virus was determined to be 5.9 × 10^5^ PFU/ml by plaque assay.

A plaque assay was performed overnight by dispensing 1.5 × 10^5^ Vero cells into 12-well plates. The next day, after confirmation of 80–100% confluency, the cells were infected with the virus for 1 h. Then, the virus was washed away, and the monolayer was overlayed with 1.5 ml of 2 × MEM supplemented with 2% cellulose (Sigma, MA, USA) (v/v 1:1).

### Samples & sampling preparation

#### Mask or respirator preparation

**Figure 1.**
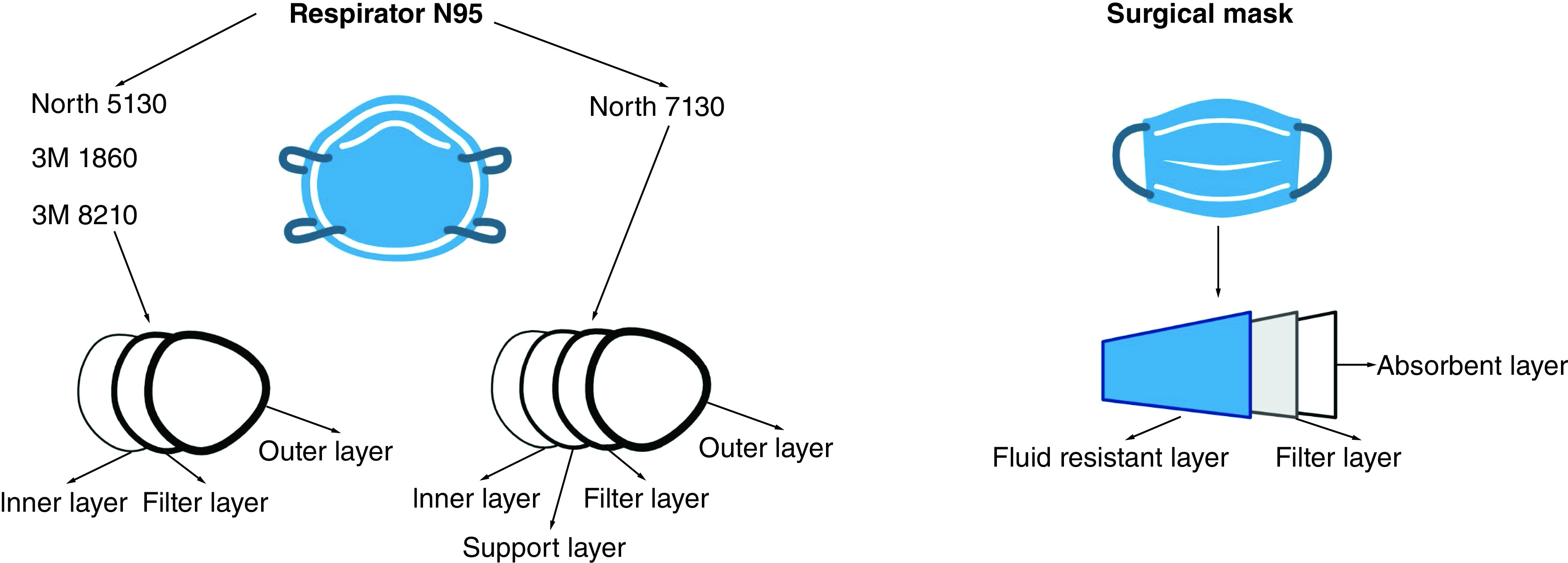
Mask or respirator layers. Description of the mask and respirator layers used in this study. Created by Biorender.com.

Five different masks or respirators (Table 1 & Figure 1) were used in this study. Pieces of 1 cm^2^ were cut, separated into individual layers, decontaminated by submerging the pieces into 70% ethanol for 10 min, and dry overnight at 37°C inside a decontaminated biosafety containment level 2. The decontaminated layers were stored in empty sterile 1.5-ml tubes until further use.

**Table 1.** Characteristics of the masks and respirators used in this study.

No.	Brand	Layer	Material	Ref.
1	North N95 7130 (lot no. 9E001) Closed-cell vinyl foam	Outer Filter Support Inner	Polypropylene Polypropylene composite Polyester Polyester	[15]
2	North N95 5130 (lot no. 9G004) Open-cell foam seal	Outer Filter Inner	Polyester Polypropylene composite Polyester	[15]
3	3-ply surgical mask	Outer Filter Inner	Fluid resistant Polypropylene Absorbent	[16]
4	3M 8210 N95 (lot no. A20047) Standard respirator	Outer Filter Inner	Polyester Polypropylene Polyester	[17]
5	3M 1860 N95 (lot no. B16047) Surgical respirator	Outer Filter Inner	Polyester Polypropylene Polyester	[17]

#### Contamination of layers

The layers were exposed to 2 × 10^3^ viruses (in 10 μl MEM). After 10 min (time to dry the drop), the layers were submerged in 300 μl of OptiMEM (Invitrogen) in a 1.5-ml screw-top tube and vortexed vigorously for 1 min. The supernatants of the samples were subjected to tenfold dilutions using OptiMEM in 1.5-ml tubes.

#### Plaque assay

Vero E6 cells were grown as detailed above. Cells were then checked for confluency, and the medium was removed from the wells. The serial dilutions (300 μl) were added to the corresponded wells, and the plates were incubated as explained above. The plates were placed back in the incubator for 1 h to allow infection of the cells. The plates were gently rocked every 15 min. Then, the virus and the medium were removed from the wells, and an overlay of 2% cellulose and 2x complete MEM (as detailed above) was gently added to each well. Plates were incubated for 72 h, fixed for 30 min with 4% p-formaldehyde (PFA, JT Baker) in phosphate-buffered saline. After removing the PFA, the cells were stained with 250 μl of 1% crystal violet (Sigma), dissolved in 20% methanol (Fisher, NH, USA) for 30 min, washed three-times with water, dried and the number of plaques was counted and recorded.

#### Statistical analysis

Statistical analysis was performed using the GraphPad Prism version 9.3.1 (350) (GraphPad, CA, USA). A p-value < 0.05 was considered significant.

## Results & discussion

The adherence of SARS-CoV-2 to different types of buccal protection showed that all of the layers could retain the virus to different extents. In addition, adherent viruses were not released upon vortexing the samples, suggesting that they are tightly bound to the layers once they are dried.

**Figure 2.**
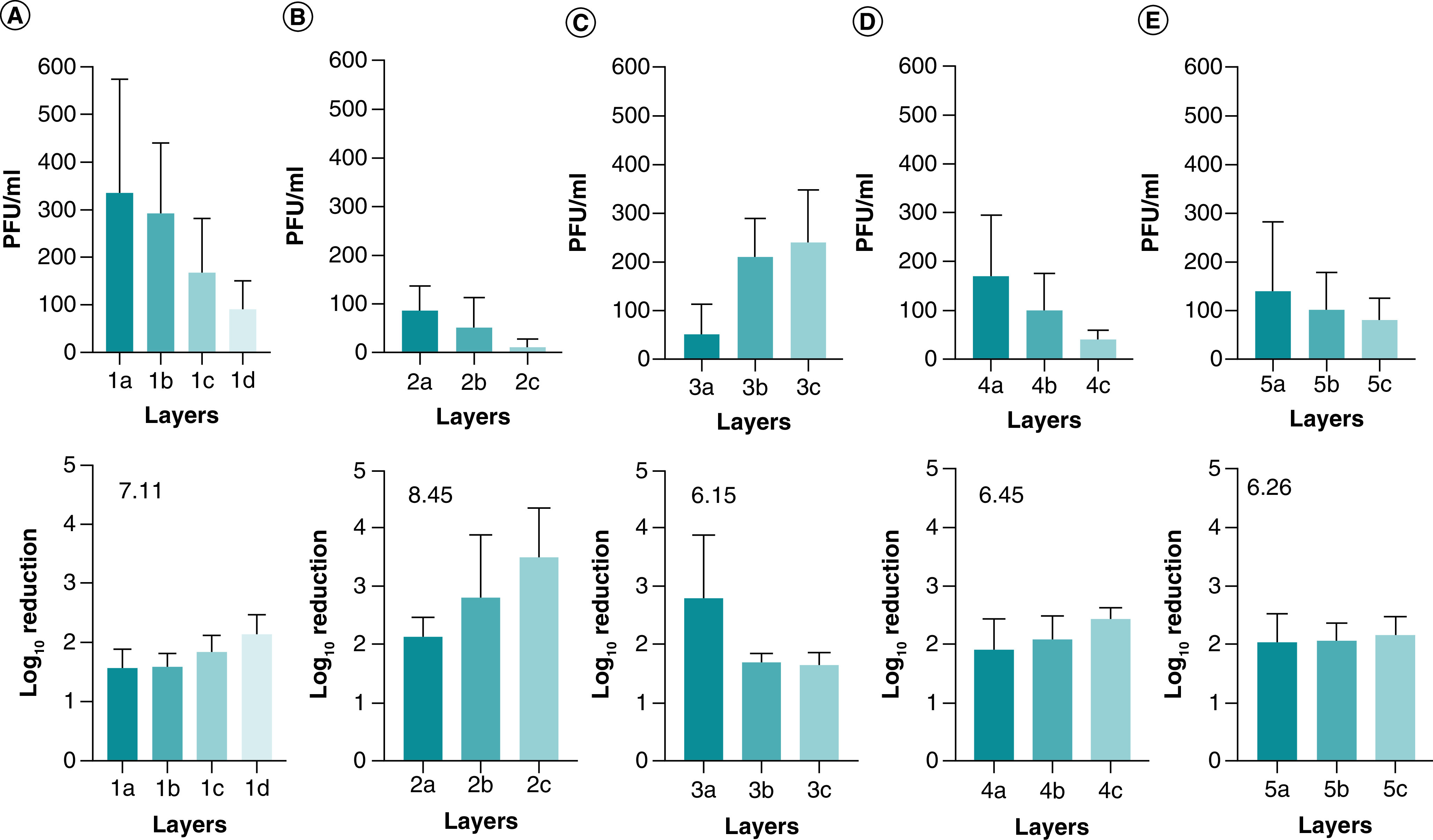
Viral titers of SARS-CoV-2 were deposited onto layers. The virus was deposited on the layers, and after drying, non-adherent viruses were released from the layers by vortexing and according to the method section. **(A)** Respirator 7130, **(B)** Respirator 5130, **(C)** Surgical masks, **(D)** Respirator 8210 and **(E)** Respirator 1860. Shown is the mean of at least three independent experiments ± SD. The number located in the lower panel is the cumulative log reduction of the layers. The arrangement of the layers is from the outer layer **(A)** to the inner layer **(C)**, except for one that has four layers **(D).** SD: Standard deviation.

Analysis of the results showed that an accumulation of the log reductions of the layers ranged between 6.15–8.45 (Figure 2). In addition, respirator 2 showed superior retention of the virus with an increase of approximately 1–2 log reduction (Figure 2B) compared with the rest of the tested products.

People use medical masks as protection from sprays and splashes of fluids by sick people. Although extended usage or reuse of masks has become common, especially during the present pandemics, this could lead to an infection of the person wearing the mask due to the possible presence of pathogens in different layers of the masks [14].

Masks materials (Table 1) are mainly non-woven fabric made by a mass of fibres (such as polypropylene, polyester and polyurethane) bonded by different means such as mechanical, heat or chemical. The efficiency of particle filtration (permeability), bacterial or virus filtration, differential pressure, flammability, flexibility and resistance to fluids are some of the performance characteristics of materials used in masks [4,5,10]. Regarding permeability, when resistance to fluid material is used, aerosols are likely to travel around the mask following contours seeking low resistance areas or leeks rather than penetrating the filter material [5].

Of the different layers in a mask or respirator, the most important one is the filter layer, which is mainly produced through a melt-blown process, where a polymer (such as polyethylene) is exposed to blows of high-velocity air, forming a web shape of filaments. This filter layer (commonly used in N95 respirators) has an electrostatic charge; therefore, the polymer filtration capability for small particles increases because of electrostatic adsorption [4,18]. In our study, the respirators and the mask tested contained at least one layer of polypropylene, suggesting that an electrostatic layer increased the adherence of the virus.

A model of viral retention based on the electrostatic charges on the surface of the virus and the modified polypropylene, termed electret, might explain one of the reasons for the log reduction of the virus.

SARS-CoV-2 is decorated by the spike protein, which is crucial for the infection of the host. Assuming that other components of the virus are not contributing to the surface charge of the virus (lipids and nucleocapsids are part of the core of the virus [19]), we can attribute the net surface charge of the virus to the spike protein. This protein looks to have a particular disorder [20], probably because of its interaction with the membrane proteins found in the virus [21]. Thus, the electrostatic interaction of the virus and the electret is based on the charge of the spike protein [22]. One of the main variables regarding the isoelectric point of the spike protein is the large number of ionizable amino acids that contribute to the net charge (e.g., C, D, E, H, K, R and Y) [19]. Then, the pH will play an important role in the fluctuation of the surface charge of the virus.

The isoelectric point of the spike protein is 6.2 [23]. Although this value is lower than the physiological pH (7.0), most spike protein is negatively charged [19], except for the region corresponding to the receptor-binding domain. This receptor is located in the apical portion of the spike protein, and it has been calculated to remain positively charged through a range of pH values [24]. Interestingly, the positive charge of the receptor-binding domain has increased in the SARS-CoV-2 variants because of the mutations observed in this region [24].

On the other hand, studies reported negative surface charges on the electrets, varying between -200 and -1350 (V) [25,26]. Thus, a strong electrostatic attraction occurs between the receptor-binding domain of the spike protein and the electret, contributing to the retention or adsorption of the viruses on the respirator or mask layers.

In conclusion, we report that the tested respirators and the medical mask could reduce at least six logs of the viral load of the SARS-CoV-2 Delta variant. This reduction was probably related to an electrostatic adherence of the virus on the tested materials. Therefore, we conclude that these results support the fact that wearing masks or respirators reduces the load of the virus either dispersed in the environment from an infected person or reducing the viral load in healthy people.

## Future perspective

This study presents the findings that facial protection reduces the number of viruses by adherence. Future studies should focus on the facial safety of other materials used to fabric masks and respirators.

**Summary points**

- The use of facial protection has been established to reduce the transmissibility of SARS-CoV-2.
- The viruses effectively adhered to the different layers of the mask or respirator.
- Facial protection may decrease the transmissibility and infection caused by SARS-CoV-2.
